# Comparison of four nanoparticle monitoring instruments relevant for occupational hygiene applications

**DOI:** 10.1186/s12995-019-0247-8

**Published:** 2019-11-27

**Authors:** Rikke Bramming Jørgensen

**Affiliations:** Department of Industrial Economics and Technology Management, Norwegian University of Science and Technology, 7491 Trondheim, Norway

**Keywords:** Ultrafine particles  (UFP), Workplace exposure, SMPS, FMPS, Nanoscan SMPS

## Abstract

**Background:**

The aim of this study is to make a comparison of a new small sized nanoparticle monitoring instrument, Nanoscan  SMPS, with more traditional large size instruments, known to be precise and accurate [Scanning Mobility Particle Sampler (SMPS) and Fast Mobility Particle Sizer (FMPS)], and with an older small size instrument with bulk measurements of 10–1000 nm particles (CPC3007). The comparisons are made during simulated exposure scenarios relevant to occupational hygiene studies.

**Methods:**

Four scenarios were investigated: metal inert gas (MIG) welding, polyvinyl chloride (PVC) welding, cooking, and candle-burning. Ratios between results are compaed and Pearsson correlations analysis was performed.

**Results:**

The highest correlation between the results is found between Nanoscan and SMPS, with Pearsson correlation coefficients above 0.9 for all scenarios. However, Nanoscan tended to overestimate the results from the SMPS; the ratio between the UFP concentrations vary between 1.44 and 2.01, and ratios of total concentrations between 1.18 and 2.33. CPC 3007 did not show comparable results with the remaining instruments.

**Conclusion:**

Based on the results of this study, the choice of measurement equipment may be crucial when evaluating measurement results against a reference value or a limit value for nanoparticle exposure. This stresses the need for method development, standardisation, and harmonisation of particle sampling protocols before reference values are introduced. Until this is established, the SMPS instruments are the most reliable for quantification of the concentrations of UFP, but in a more practical occupational hygiene context, the Nanoscan SMPS should be further tested.

## Introduction

Exposure assessment of nanoparticle exposure is performed in a wide range of occupational situations, from traditional industries, such as the ferroalloy, silicon alloy, and silicon carbide industries [1–3], industrial plants with activities, such as fettling, laser cutting, welding, smelting, core making, moulding, concreting, grinding, and sieving of powders [4], to construction sites [5, 6], restaurants [7], and nanotechnology workplaces [8–10].

Personal sampling is well established as a sampling method for occupational exposure assessment. However, the technical solutions for nanoparticle measurement are not suitable for traditional personal sampling with pumps, cyclones, and filter cassettes, and the major part of the studies performed are made with direct-reading stationary monitors. Only a few personal sampling studies have been performed [5, 7, 11, 12]. This is due to the fact that the equipment for nanoparticle measurement have until now been large and power demanding, where the only way to undertake personal sampling was by the use of a sampling tube, and then, for short measurement periods or completely stationary workplaces. Some smaller equipment has been on the market for a while now, which measure a larger fraction of submicrometre particles. Some examples are the CPC3007 from TSI, measuring a size range of 10–1000 nm; Ptrak® from TSI, measuring a size range of 20–1000 nm, the Nanotracer from Philips, measuring a size range of 10–300 nm, and the Nanocheck from GRIM (30–300 nm). This smaller equipment has been used for quasi-personal sampling using an assistant who holds the equipment close to a worker for a short period [13, 14].

The large power demanding nanoparticle instruments provide us with detailed analyses of the particle size distribution, the dominating mode of particle sizes, and enable us to build knowledge about how the particle size distributions change as a function of time. They make possible the calculation of the exact particle fraction below 100 nm, even though none of them measure diameters as low as exactly 0 nm. The small equipment produce results as a bulk measurement, in the concentration range of 10–1000 nm, and, as pointed out by Viitanen, et al. [2017] these two types of results are not directly comparable, even though both types of results are often mentioned as a concentration of ultrafine particles (UFP) [15].

The particle size distributions differ between occupational processes; some processes mainly produce particles in the submicrometre range, such as cooking fresh bacon on an electric stove, where only 35% of the particles form the ultrafine fraction [11]. PVC welding, in contrast, has 97–99% of the produced particles below 100 nm in diameter [5], and thus, in the ultrafine fraction. This difference is important when you want or need to compare the results with a threshold value defined for a certain fraction of particles. Exposure limits for workplace exposure to nanoparticles have been proposed by Broekhuizen, et al. [16] and Schutte [17], and these are made for the diameter range of 0–100 nm particles.

The difference between personal and stationary measurement is another part of the challenge. Koponen, et al. divided a factory into near-field and far-field, and studied the exposure to ultrafine particles in both situations [18]. The near-field was defined as the area 0.5 m from the powder pouring openings of the mixing tanks, which should be the most polluted operation, and the far-field as the area 5 m - 15 m from the near-field instruments in areas where there was at least 2 m distance to the area of activity. The instruments were placed in both places, and the results showed that personal level exposure was higher than that measured from the near-field. Koponen, et al. concluded that, “to better understand the activities leading to worker’s personal exposure, workers should be equipped with real-time particle monitors so that the exposure can be linked to different work tasks” [18]. This is confirmed in two reviews [5, 19], which state that there are large differences between personal samples of UFP in the breathing zone of the worker and more stationary values in work area measurements. In the evaluation of worker exposure, it is important to distinguish between the two types of measurement. In summary, there is a need for a portable instrument measuring the particle size distribution, or at least the exact ultrafine fraction below 100 nm, in the breathing zone of workers.

Comparison studies between various real-time nanoparticle monitoring instruments are mainly performed by the use of ambient aerosols [20], with fuelled vehicle emission [21, 22], or with laboratory generated particles, such as NaCl, Ag, polystyrene latex, ammonium sulphate (NH_4_)_2_SO_4_ particles, di-ethyl hexyl sebacate (DEHS) C_26_H_50_O_4_, TiO_2_, and diesel soot particles [23–26]. Only a few comparisons have been made with aerosols in the workplace [2, 27]. None of these studies have used the TSI Nanoscan  SMPS instrument, which is a small, battery-operated instrument, that may be quite useful in occupational hygiene studies. Viitanen, et al. [2017] have conducted a literature review of workplace measurements of UFP and point out that particle number concentration in various studies are not fully comparable and require parallel measurements using different techniques to improve the evaluation of the comparability of past studies [15].

The aim of this study is to make a comparison of a new small sized instrument, Nanoscan SMPS, which is battery-operated and measures the particle size distribution of particles in the size range of 10–421.7 nm, with more traditional large instruments known to be precise and accurate (SMPS and FMPS) and with an older small size instrument, which takes bulk measurements (CPC3007). The comparisons are made with exposure scenarios relevant to occupational hygiene studies: MIG (MIC) welding, polyvinyl chloride (PVC) welding, cooking, and candle-burning.

## Methods

### Exposure scenario

Four different exposure situations were defined, and laboratory setups were made for investigation of the particle emission/exposure from each of them:

#### Welding fume by use of a MIC welding robot

A welding robot placed in a small closed room performed the MIC welding. The welding was performed continuously for 1 min and 30 s. After each welding period, the welding sheet was turned manually to a new area and the welding was restarted. Every new welding period started 2 min after the start of the previous period. Seven repetitions were performed, in total 10.5 min of welding. Thereafter, the concentration in the room was measured for 30 min before the room was ventilated with forced ventilation for 15 min, until the concentration of fumes in the room returned to the background concentration. Five repetitions of the 10.5 min welding procedure were performed.

#### Welding fume by PVC welding

PVC welding was performed on the floor in a small room. A 3.03 m^2^ PVC floor was welded with welding wires of 2 m length by use of a hot air PVC welding gun. In total 23 lengths closely adjacent to one another were welded. The welding was performed manually.

#### Cooking fume

The cooking procedure is described in detail by Sjaastad [11]. In this experiment, two pieces of pork chop were used for each cooking episode (350 g). The frying pan was heated on a gas stove using maximum heat for 2 min and afterwards 15 mL of oil was added and heated for 1 min. After heating the oil, two pieces of pork chop were added to the pan, each of them ~ 165 g. The pork shops were left for 2 min and were then turned. At 3 min, the heat was reduced to medium and after 4 min the pork chops were turned for the second time. After this, the meat was turned once a minute for four minutes. Then the heat was turned off, and the meat was left in the frying pan for an additional 2 min before the pork chops were removed from the frying pan. After 2 min, forced ventilation was turned on and the laboratory was ventilated for 30 min. Three repetitions of the cooking procedure were performed.

#### Candle-burning

Four candles of 100% paraffin wax, height 10 cm, diameter 7 cm, weight 340 g, were placed on a table in a rectangle with 2 cm between each candle. The candles were lit by use of two matches in rapid succession, were burned for 4 h and were then put out in rapid succession.

### Instruments in the study

Four different instruments were used in the study. Instrument information is given in Table 1. A sampling rack with all instruments placed side by side was arranged. The samples from the instruments were drawn through flexible silicone tubes (TSI Conductive Silicone Tubing), The tubes were 1/4-in for SMPS, CPC and Nanoscan, while 3/8-in for FMPS for optimal fits to the impactors and inlets. The length of the tubes was identical for alle instruments (0.6 m). The tubes were assembled with a band ensuring that the aerosol collection took place at the same place. Measurements with all instruments were performed simultaneously, and the inlet of the tubings was placed as close as possible to the particle source.

**Table 1:** Instrument information

	Size (m)	Weight (kg)	Power demands	Time resolution	Liquid	Diameter range (nm)	Studied metric	Principal particle size	Aerosol flow rate (lpm)	Sheath flow (lpm)	Aerosol Charger
SMPS	0.41 · 0.28 · 0.65 + 0.25 · 0.32 · 0.37	30	Power	60 s	Butanol	13.3–487	PNC + PSD	Electrical mobilty	0.3	4	Bipolar ^85^Kr neutralizer
FMPS	0.7 · 0.34 · 0.44	34	Power	1 s *	–	5.6–560	PNC + PSD	Electrical mobilty	10	40	Unipolar diffusion charger (corona needle)
Nanoscan	0.45 · 0.23 · 0.39	8	Battery or Power	60 s	Isopropanol	10–420	PNC + PSD	Electrical mobilty	0.75	–	Non-radioactive unipoar diffusion charger (corona jet type)
CPC	0.53 · 0.36 · 0.21	1.7	Battery	60 s	Isopropanol	10–1000	PNC	–	0.67	–	–

PNC: Particle Number Concentration PSD: Particle Size Distribution *: calculated as mean results during  60 s

#### SMPS

A Scanning Mobility Particle Sizer (model 3938 L76, TSI Incorporated, Shoreview, USA) was used in the study, hereafter called SMPS. The SMPS consists of an electrostatic classifier (EC, model 3082), a long differential mobility analyser (DMA model 3081A) and an ultrafine condensation particle counter (CPC model 3736) which is butanol based.

The following settings were used: the electrostatic classifier (EC) was fitted with a 0.0457 cm impactor nozzle with a flow rate of 0.3 L/min and a sheath flow rate of 4.0 L/min. Scanning time: 60 s, retrance time 4 s and purge time 10 s; which gives a total time of 74 s for each measurement. Repeated measurements were performed every 2 min. This setting corresponds to a measurement range of 13.3 nm to 487 nm. The samples were drawn through a flexible silicone tube (TSI Conductive Silicone Tubing). This tube fits the impactor of the EC optimally (1/4-in). The length of the tube was 0.6 m. The results are presented with multiple charge correction and diffusion correction.

#### FMPS

Fast Mobility Particle Sizer (FMPS, model 3091, TSI, Shoreview, MN, USA) was used for the experiments together with a dilutor with a dilution rate 100:1 (DIL 550, TOPAS GmbH, Germany. The size range and number of channels is described in Table 2; the structure and use of the instrument is described before [5, 28].

**Table 2:** Instrument information of particle size ranges

	Particle size range of the instrument (nm)	Channels	Range of UFP (nm)	Range of 100–200 nm	Range of total particle concentration (nm)
SMPS	13.3–487	64	13.3–99.98	99.99–198.1	13.3–487
FMPS	5.6–560	32	5.6–100.04	100.05–205	5.6–560
Nanoscan	10–420	13	9.93–100.01	100.02–177	10–420
CPC	10–1000	1	–	–	10–1000

#### CPC 3007

A handheld condensation particle counter (CPC, model 3007, TSI, Shoreview, MN, USA) was used in the study, hereafter mentionen CPC. The instrument and the use of it is described before [2]. Each measurements were logged with an averaging period of 1 min. Each measurement period had a maximum duration of 2 h before the CPC was loaded with alcohol and restarted.

#### Nanoscan SMPS

A NanoScan SMPS (model 3910, TSI, Shoreview, MN, USA) was used in the study. The instrument operates at a flow rate of 0.75 L/min. After the inlet, the aerosol flow passes a cyclone with a cut point (D50) of 550 nm to remove larger particles. Then the polydiserse aerosol enters a unipolar charger. This unipolar charger works through the introduction of an opposed charger flow filtered with an active carbon filter and a HEPA filter. After passing over the charger needle, a jet of positive ions flows and enters a mixing chamber, allowing the interaction of ions with the aerosol sample using the opposed flow technique. After passing the charging region, the sample flow enters the radial DMA where the particles are classified based on their electrical mobility in the rDMA. The size classified monodisperse aerosols exit the rDMA according to the strength of the electric field and are finally measured at an isopropanol-based CPC. During the course of a measurement the DMA‘s voltage scans the entire size range, which for the Nanoscan SMPS is 10 nm to 421.7 nm in 13 channels.

### Data analysis

Each instrument was operated by a personal computer with the software belonging to the instrument. NanoScan Manager software (TSI, version 1.0.0.19); Aerosol Instrument Manager software (TSI, version 10.2) and Fast Mobility Particle Sizer Software (TSI model 3.1.1.0).

The results in this study are calculated, reported and compared in three fractions: as concentrations of particles < 100 nm, mentioned *UFP*, as the concentration of particles in the total range of the instrument, mentioned *total* and as the concentration of particles in the 100–200 nm range, mentioned *100–200 nm.* Table 1 shows the exact particle sizes for each instrument within these fractions.

The results are calculated as arithmetic mean (AM) with corresponding standard deviation (SD) geometric mean (GM)) with corresponding geometric standard deviation (GSD), and reported together with minimum, maximum, median, and interquartile range (IQR) values. In order to compare the AM values of each instruments against the SMPS results, the ratio between the UFP concentration, the total concentration and the 100–200 nm concentration is calculated.

Correlations between different measurement are evaluated using the Pearsson correlation coefficients. 

All particle diameters correspond to the electrical mobility diameter as measured by the instruments. The particle size distribution is reported as normalised concentration (dNdlogDp) as described by TSI [29], because this conversion allows comparison with data from other instruments.

## Results

### Particle number concentrations results

The descriptive statistics of all experiments are shown in Table 3. The results for the welding and cooking experiments are calculated from the data showing the activity without the periods between that were without activity, however, did have ventilation. For the PVC welding and candle-burning experiments, the start-up of the experiment and the decaying concentration after finishing the activity are not included.

**Table 3:** Descriptive statistics for the different exposure scenarios and the different instruments

			PVC welding *N* = 25	Candle-burning *N* = 125	MIC welding *N* = 45	Cooking *N* = 32
			#/cm^3^	#/cm^3^	#/cm^3^	#/cm^3^
UFP	SMPS	AM^a^	5.23 × 10^5^	5.24 × 10^4^	3.79 × 10^4^	5.37 × 10^5^
		SD^b^	3.78 × 10^5^	7.79 × 10^3^	2.83 × 10^4^	3.33 × 10^5^
		GM^c^	4.31 × 10^5^	5.18 × 104	2.76 × 10^4^	3.22 × 10^5^
		GSD^d^	1.86	1.18	2.48	4.17
		Min^e^	1.36 × 10^5^	2.76 × 10^4^	2.32 × 10^3^	5.97 × 10^3^
		Max^f^	1.71 × 10^6^	6.97 × 10^4^	1.46 × 10^5^	9.72 × 10^5^
		Median	4.48 × 10^5^	5.24 × 10^4^	3.54 × 10^4^	5.73 × 10^5^
		IQR^g^	2.21 × 10^5^	9.52 × 10^3^	2.54 × 104	5.86 × 10^5^
	FMPS	AM	3.93 × 10^5^	1.74 × 10^5^	1.00 × 10^5^	4.46 × 10^5^
		SD	1.77 × 10^5^	1.89 × 10^4^	3.37 × 10^4^	2.45 × 10^5^
		GM	3.62 × 10^5^	1.72 × 10^5^	9.39 × 10^4^	3.33 × 10^5^
		GSD	1.50	1.14	1.48	2.61
		min	1.62 × 10^5^	8.28 × 10^4^	2.18 × 10^4^	3.42 × 10^4^
		max	9.13 × 10^5^	2.33 × 10^5^	1.95 × 10^5^	8.00 × 10^5^
		median	3.70 × 10^5^	1.77 × 10^5^	9.65 × 10^4^	4.72 × 10^5^
		IQR	8.73 × 10^4^	1.34 × 10^4^	4.37 × 10^4^	3.59 × 10^5^
	Nanoscan	AM	7.55 × 10^5^	8.11 × 10^4^	7.64 × 10^4^	8.47 × 10^5^
		SD	3.25 × 10^5^	1.02 × 10^4^	3.97 × 10^4^	5.70 × 10^5^
		GM	6.84 × 10^5^	8.03 × 10^4^	6.79 × 10^4^	4.24 × 10^5^
		GSD	1.63	1.15	1.85	5.29
		min	1.42 × 10^5^	4.67 × 10^4^	1.08 × 10^4^	6.19 × 10^3^
		max	1.56 × 10^6^	1.13 × 10^5^	1.84 × 10^5^	1.62 × 10^6^
		median	7.27 × 10^5^	8.13 × 10^4^	7.49 × 10^4^	1.12 × 10^6^
		IQR	2.23 × 10^5^	8.71 × 10^3^	4.34 × 10^4^	1.05 × 10^6^
Total	SMPS	AM	1.04 × 10^6^	5.29 × 10^4^	1.59 × 10^5^	9.19 × 10^5^
		SD	3.42 × 10^5^	7.53 × 10^3^	1.03 × 10^5^	6.11 × 10^5^
		GM	9.66 × 10^5^	5.23 × 10^4^	9.87 × 10^4^	5.12 × 10^5^
		GSD	1.58 × 10^5^	1.17 × 10^5^	3.66	4.61
		min	1.48 × 10^5^	2.92 × 10^4^	3.08 × 10^3^	6.53 × 10^3^
		max	2.07 × 10^6^	6.99 × 10^4^	3.54 × 10^5^	1.92 × 10^6^
		median	1.02 × 10^6^	5.27 × 10^4^	1.61 × 10^5^	1.05 × 10^6^
		IQR	1.93 × 10^5^	9.43 × 10^3^	1.28 × 10^5^	1.04 × 10^6^
	FMPS	AM	5.57 × 10^5^	1.75 × 10^5^	4.38 × 10^5^	5.86 × 10^5^
		SD	1.59 × 10^5^	1.89 × 10^4^	2.78 × 10^5^	3.55 × 10^5^
		GM	5.34 × 10^5^	1.74 × 10^5^	3.23 × 10^5^	4.08 × 10^5^
		GSD	1.37	1.14	2.54	2.94
		min	1.84 × 10^5^	8.51 × 10^4^	2.18 × 10^4^	3.42 × 10^4^
		max	9.70 × 10^5^	2.35 × 10^5^	9.86 × 10^5^	1.14 × 10^6^
		median	5.56 × 10^5^	1.78 × 10^5^	3.95 × 10^5^	6.21 × 10^5^
		IQR	9.21 × 10^4^	1.29 × 10^4^	3.75 × 10^5^	5.41 × 10^5^
	Nanoscan	AM	1.22 × 10^6^	8.55 × 10^4^	3.71 × 10^5^	1.14 × 10^6^
		SD	3.22 × 10^5^	1.05 × 10^4^	2.23 × 10^5^	8.08 × 10^5^
		GM	1.14 × 10^6^	8.48 × 10^4^	2.83 × 10^5^	5.50 × 10^5^
		GSD	1.59	1.15	2.44	5.40
		min	1.50 × 10^5^	4.98 × 10^4^	1.72 × 10^4^	6.83 × 10^3^
		max	1.82 × 10^6^	1.19 × 10^5^	8.87 × 10^5^	2.40 × 10^6^
		median	1.25 × 10^6^	8.57 × 10^4^	3.26 × 10^5^	1.47 × 10^6^
		IQR	1.89 × 10^5^	8.96 × 10^3^	3.09 × 10^5^	1.48 × 10^6^
	CPC	AM	5.41 × 10^5^	1.55 × 10^5^	2.30 × 10^5^	4.12 × 10^5^
		SD	9.10 × 10^4^	1.50 × 10^4^	1.21 × 10^5^	1.93 × 10^5^
		GM	5.30 × 10^5^	1.54 × 10^5^	1.74 × 10^5^	3.09 × 10^5^
		GSD	1.27	1.13	2.63	2.75
		min	2.16 × 10^5^	8.54 × 10^4^	5.24 × 10^3^	2.52 × 10^4^
		max	5.82 × 10^5^	1.68 × 10^5^	4.53 × 10^5^	6.11 × 10^5^
		median	5.70 × 10^5^	1.59 × 10^5^	2.26 × 10^5^	5.14 × 10^5^
		IQR	1.14 × 10^4^	6.76 × 10^3^	1.52 × 10^5^	1.93 × 10^5^
100–200 nm	SMPS	AM	3.98 × 10^5^	3.59 × 10^2^	2.41 × 10^4^	2.96 × 10^5^
		SD	1.16 × 10^5^	2.52 × 10^2^	1.60 × 10^4^	2.69 × 10^5^
		GM	3.07 × 10^5^	3.08 × 10^2^	1.37 × 10^4^	1.02 × 10^5^
		GSD	3.90	1.68	4.37	7.90
		min	5.24 × 10^2^	1.20 × 10^2^	3.12 × 10^2^	3.91 × 10^2^
		max	5.43 × 10^5^	1.59 × 10^3^	5.51 × 10^4^	7.99 × 10^5^
		median	4.14 × 10^5^	2.95 × 10^2^	2.35 × 10^4^	2.34 × 10^5^
		IQR	6.24 × 10^4^	1.60 × 10^2^	2.63 × 10^4^	4.47 × 10^5^
	FMPS	AM	1.45 × 10^5^	1.07 × 10^3^	2.23 × 10^5^	1.29 × 10^5^
		SD	4.68 × 104	2.80 × 10^2^	1.76 × 10^5^	1.28 × 10^5^
		GM	1.24 × 10^5^	1.03 × 10^3^	5.43 × 10^4^	2.38 × 10^4^
		GSD	2.30	1.33	32.53	18.74
		min	2.94 × 10^3^	3.90 × 10^2^	5.00 × 10^0^	1.00 × 10^2^
		max	2.20 × 10^5^	1.79 × 10^3^	5.60 × 10^5^	3.86 × 10^5^
		median	1.61 × 10^5^	1.05 × 10^3^	1.94 × 10^5^	8.65 × 10^4^
		IQR	5.17 × 104	3.88 × 10^2^	2.32 × 10^5^	2.28 × 10^5^
	Nanoscan	AM	4.32 × 10^5^	3.56 × 10^3^	1.37 × 10^5^	2.63 × 10^5^
		SD	1.42 × 10^5^	4.44 × 10^2^	1.01 × 10^5^	2.60 × 10^5^
		GM	3.61 × 10^5^	3.53 × 10^3^	8.16 × 10^4^	7.33 × 10^4^
		GSD	2.54	1.14	3.94	8.65
		min	5.76 × 10^3^	2.23 × 10^3^	1.36 × 10^3^	5.96 × 10^2^
		max	5.97 × 10^5^	4.86 × 10^3^	3.70 × 10^5^	7.52 × 10^5^
		median	4.78 × 10^5^	3.51 × 10^3^	1.22 × 10^5^	2.65 × 10^5^
		IQR	9.41 × 10^4^	5.44 × 10^2^	1.41 × 10^5^	4.96 × 10^5^

a) Arithmetric mean b) standard deviation c) geometric mean d) geometric standard deviation e) minimum concentration f) maximum concentration, g) interquartile range h) number of measurements

The results show large variations in measured concentrations between the instruments and scenarios. PVC welding, cooking, and candle-burning are the scenarios showing the highest concentrations of UFP, with an AM at 1.74 × 10^5^–8.47 × 10^5^ particles/cm^3^, while MIC welding shows the lowest UFP concentrations with an AM at 3.79 × 10^4^ - 1.00 × 10^5^ particles/cm^3^. Both AM and GM are reported and large variations between the AM and GM values are seen, especially, for the experiments with high IQR.

The ratio between the different instruments and the SMPS results is shown in Table 4. The ratio between Nanoscan and SMPS is 1.44–2.01, while the FMPS instrument shows both higher and lower concentrations. For the candle-burning scenario, the FMPS showed 3.31 times as high concentration as SMPS, while for the PVC welding scenario, the FMPS showed lower concentrations than SMPS, with a ratio of 0.75.

**Table 4:** Ratio between concentrations measured by the mentioned instruments and the SMPS

		PVC welding	Candle-burning	MIC welding	Cooking
UFP	FMPS	0.75	3.31	2.65	0.83
	Nanoscan	1.44	1.55	2.01	1.58
	CPC	1.04	2.96	6.05	0.77
total	FMPS	0.54	3.31	2.75	0.64
	Nanoscan	1.18	1.62	2.33	1.24
	CPC	0.52	2.93	1.44	0.45
100–200 nm	FMPS	0.36	2.98	9.29	0.44
	Nanoscan	1.09	9.90	5.70	0.89
	CPC	1.36	431	9.54	1.39

The CPC instrument measures a bulk range of 10–1000 nm, but is often referred to as a nano/UFP measurement. The CPC results comply with SMPS results for UFP in the PVC welding scenario (ratio 1.04), but was closest to the total concentration for the MIC scenario (ratio 1.44) and to the 100–200 nm concentration for the cooking scenario, while the candle-burning scenario showed a very high ratio between CPC and SMPS (ratio 2.93–431).

### Particle size distribution

**Fig. 1:**
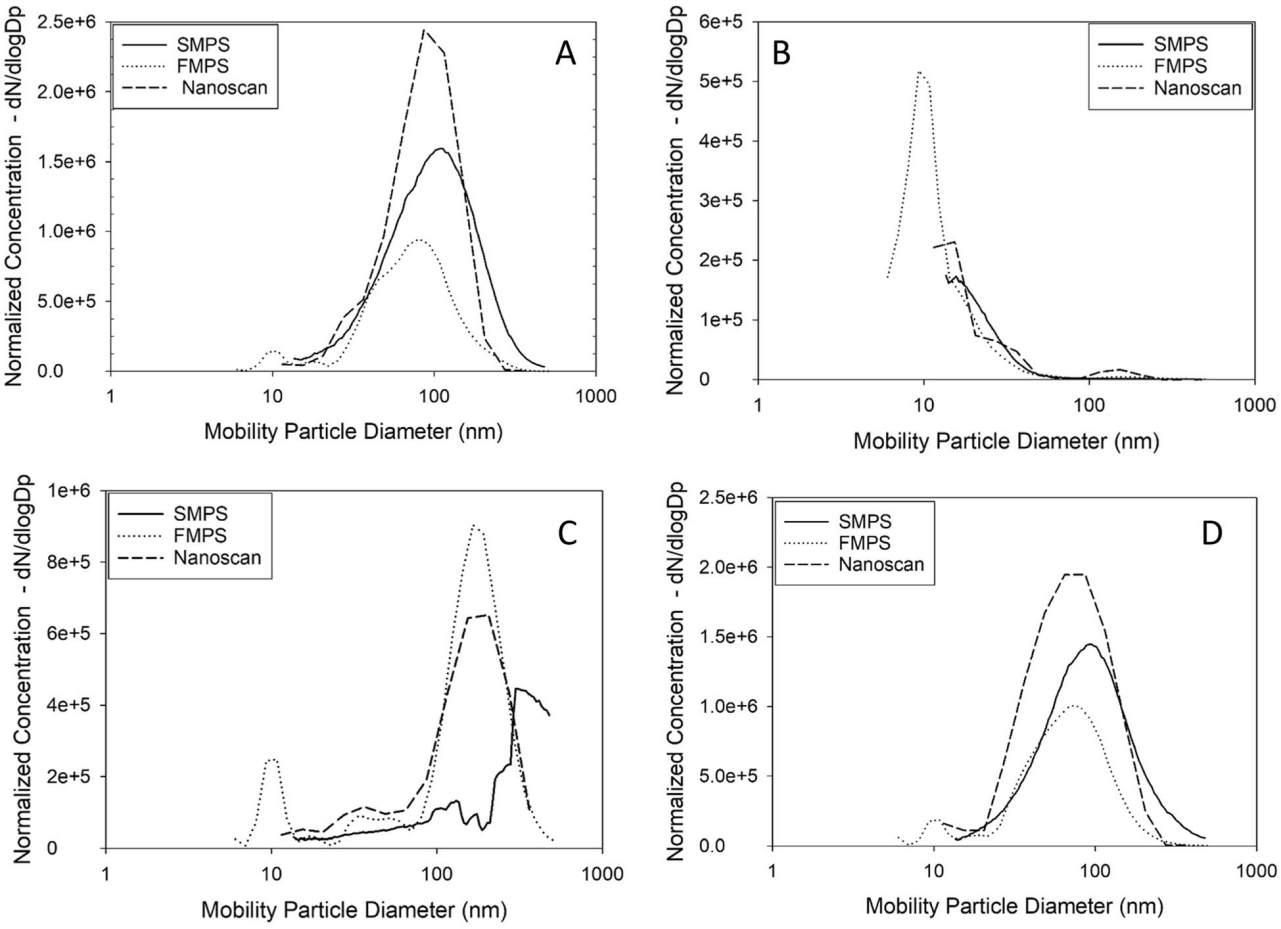
Particle size distribution during four different scenarios measured with SMPS, FMPS and Nanoscan. **a**: PVC welding, **b**: Candle-burning, **c**: MIC welding and **d**: Cooking

Figure 1 shows the particle size distributions calculated as the mean size distribution during each scenario for FMPS, SMPS, and Nanoscan. For the PVC welding and cooking scenarios, the three instruments show the same particle distributions. For the candle-burning scenario, the FMPS measurements found that the highest emissions occur for particles with a diameter as low as 9.31 nm. This is below the size range of both the Nanoscan and the SMPS (at least with the setup used for this experiment). The difference in the particle size distribution is reflected in the results in Table 3, where the AM and GM for the FMPS were higher than the results from the SMPS and Nanoscan instruments.

During MIC welding, the SMPS instrument found larger particles compared to both the Nanoscan and FMPS. The dominating mode for the SMPS was 310 nm, with high levels also in the size range from 310 to 478 nm, but a low level at lower particle sizes, while the Nanoscan showed a peak at 205 nm and the FMPS instrument showed a two-peak size distribution with a clear peak at 165 nm and a smaller peak at 10.8 nm. This also explains some of the differences in the AM/GM values.

### Concentration versus time profiles

**Fig. 2:**
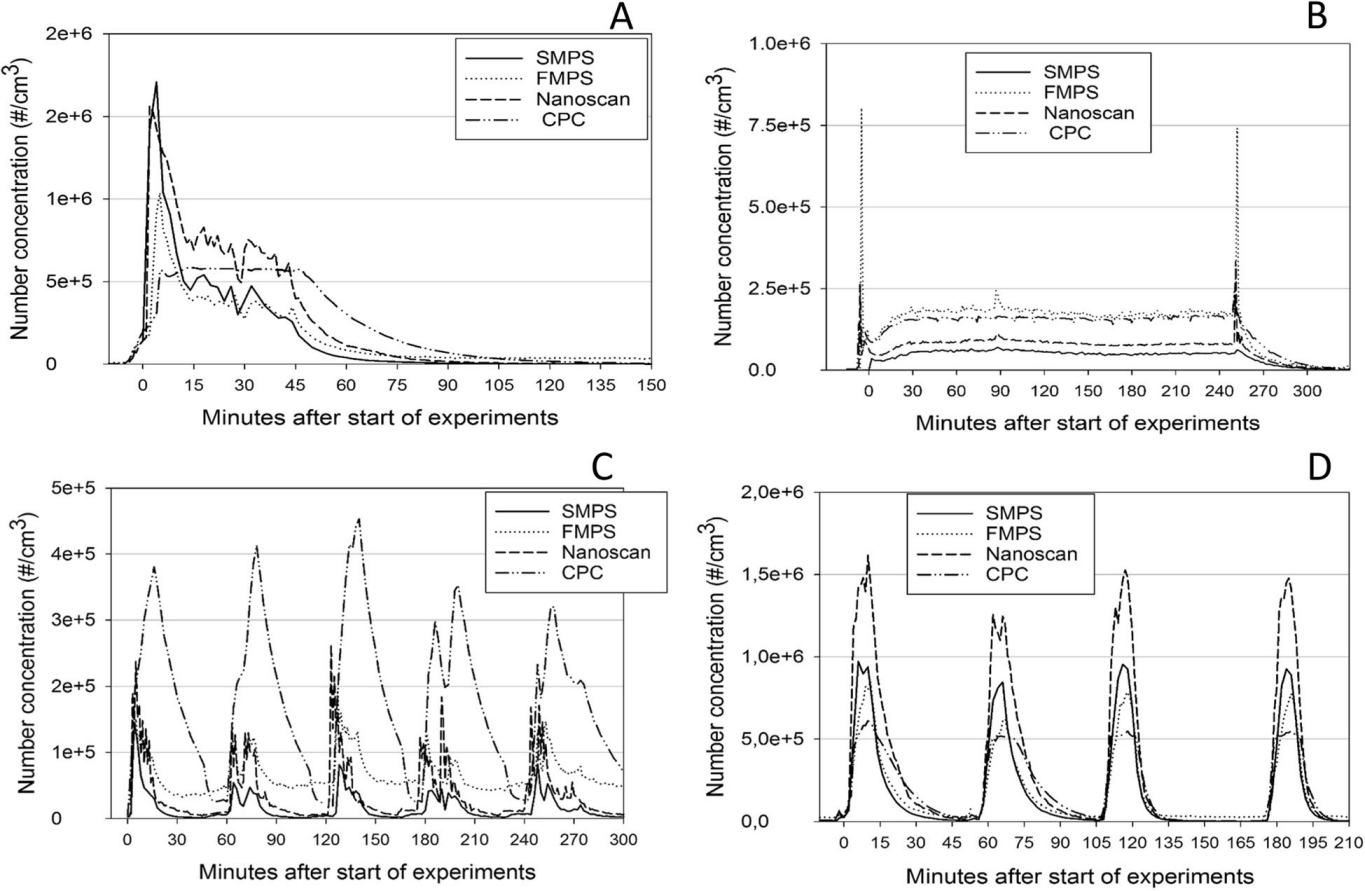
Particle number concentration of UFP as function of time during four different scenarios measured with SMPS, FMPS, Nanoscan and CPC3007. **A**: PVC welding, **B**: Candle-burning, **C**: MIC welding and **D**: Cooking

Figure 2 shows the nanoparticle concentration versus time profile of the four scenarios. Here, the measurements are shown as the experiments were performed, including the periods with no activity for the MIC welding and cooking experiments (the decay periods). For the candle-burning scenario, Fig. 2 includes a short period of lighting the candles with two matches, putting them out at the end of the experiment and allowing for decay, which not is included in the calculations.

As seen in Fig. 2, the concentration patterns differ between the scenarios; this is mainly caused by differences between the experimental designs. The decay of the CPC measurements after the end of experiments seems to be slower than the decay of the remaining instruments. The FMPS instrument shows a build-up in concentration during the experiment for the MIC welding; the design of the experiment was planned to result in a decrease in concentration back to background level between each welding period, but neither the FMPS nor the CPC reached the background level between the experiments.

Table 5 shows the correlation statistics between the various instruments used during the measurements, as illustrated in Fig. 2, and calculated using Pearsson correlation statistics. Most of the correlations are significant, as indicated with ^**^ after the results. For the UFP results, the best correlation is found between the FMPS, SMPS, and Nanoscan for the PVC welding and cooking scenarios (perfect correlations defined as Pearsson correlation coefficient = ± 1). For the CPC, the only correlation in the UFP range that is comparable to the correlation for the SMPS/FMPS/Nanoscan instruments are the concentration versus time results of UFP for the cooking scenario.

**Table 5:** Pearson correlations coefficients

Size range	Instrument	FMPS	Nanoscan	CPC
**PVC welding**				
UFP	SMPS	0,968^**^	0,963^**^ 0.980^**^	-0,199
	FMPS			-0,243
	Nanoscan			−0.633^**^
total	SMPS	0,934^**^	0,951^**^ 0.916^**^	0,213
	FMPS			0.09
	Nanoscan			−0.468^*^
100–200 nm	SMPS	0,960^**^	0,986^**^	0,959^**^
	FMPS		0,974^**^	0,965^**^
	Nanoscan			0,959^**^
**Candle-burning**				
UFP	SMPS	0,828^**^	0,942^**^	0,720^**^
	FMPS		0,896^**^	0,827^**^
	Nanoscan			0.778^**^
total	SMPS	0,818^**^	0,933^**^	0,699^**^
	FMPS		0,894^**^	0,820^**^
	Nanoscan			0.770^**^
100–200 nm	SMPS	0,499^**^	−0,381^**^	−0,873^**^
	FMPS		−0,052	−0,520
	Nanoscan			0,318^**^
**MIC welding**				
UFP	SMPS	0,734^**^	0,911^**^	0,514^**^
	FMPS		0,756^**^	0,492^**^
	Nanoscan			0,602^**^
total	SMPS	0,974^**^	0,982^**^	0,964^**^
	FMPS		0,976^**^	0,961^**^
	Nanoscan			0,952^**^
100–200 nm	SMPS	0,912^**^	0,909^**^	0,9176^**^
	FMPS		0,966^**^	0,956^**^
	Nanoscan			0,930^**^
**Cooking**				
UFP	SMPS	0,977^**^	0,987^**^	0,891^**^
	FMPS		0,985^**^	0,925^**^
	Nanoscan			0,930^**^
total	SMPS	0,994^**^	0,990^**^	0,924^**^
	FMPS		0,981^**^	0,926^**^
	Nanoscan			0,950^**^
100–200 nm	SMPS	0,968^**^	0,971^**^	0,837^**^
	FMPS		0,950^**^	0,789^**^
	Nanoscan			0,838^**^

## Discussion

### Particle number concentration results

The SMPS and FMPS are often used for studies of UFP, while the Nanoscan is a new instrument, with only a few reported studies [29–32]. Comparing the SMPS and Nanoscan results of UFP, Table 3 shows that Nanoscan consequently shows higher UFP concentrations than SMPS. Moreover, the ratio between the UFP results (Table 4) vary between 1.44 and 2.01 and the total concentration between 1.18 and 2.33. Stabile, et al. [2014] used the Nanoscan SMPS for metrological assessments and found that the Nanoscan overestimates the number concentration in the case of fresh aerosols compared to the SMPS [30].

One of the experiments done by Stabile, a grill experiment, is directly comparable to the cooking experiment in this study. Stabile found a ratio between the Nanoscan results and the SMPS results of 0.96, with diffusion and a multiple charge correction (1.33 without correction) performed as the ratio between the total concentrations measured [30]. The results in this study show a ratio between these two results of 1.24, as shown in Table 4. When comparing all scenarios, our results support the results from Stabile, where Nanoscan shows higher concentrations than the SMPS. The highest ratio found was in the MIC scenario. Fonseca, et al. [2016], who found that Nanoscan tends to overestimate particle concentrations, particularly for agglomerated particles, also observed this type of result [33].

The concentrations measured by the FMPS changed between lower concentrations more than SMPS/Nanoscan for the PVC welding and cooking scenarios, while the concentrations were higher than SMPS/Nanoscan for the MIC welding and candle-burning scenarios. Most of the comparisons performed between FMPS and SMPS instruments were performed with standardised test aerosols, like NaCl and diesel soot [24], TiO_2_, and Amoniumsulphate (NH_4_)_2_SO_4,_ [23]_,_ and shows that the type of aerosol influence the results, especially the morphology of the particles seems to influence the FMPS results by influencing the charging of the particles.

For the FMPS, the particle number concentration is determined by measurement of the electrical current collected on a series of electrodes followed by an inversion algorithm to convert currents to concentrations. This algorithm is developed by the use of an SMPS instrument and handles the time delay between electrometers, multiple voltage on the centre rod, multiple charges, among other things. It has been pointed out, that the FMPS instrument measures a smaller particle size and higher concentrations compared to SMPS, while measuring agglomerates [23, 24]; however, whether this is due to the calibration of the instruments, which is done by spherical particles, or the differences in charging system between the instruments is unknown.

Viitanen, et al. [2017] point out that there is disagreement in the literature about how to investigate and report exposure and emission of nanoparticles [15]. For example, Viitanen, et al. mention that UFP studies are presented based on results measured with instruments whose measurement ranges are not limited by the UFP definition [15]. Occupational exposure limits for nanomaterials and nanoparticles have been discussed [16, 17] and a set of ‘nano reference values’ (NRV) are proposed [34]. These values are given as the number concentration of nanoparticles, which means the number concentrations of particles is < 100 nm. To make a proper risk evaluation of worker exposure in companies and a proper evaluation of the effect of implemented measures, it is important to establish knowledge about the relationship between the results from instruments like the CPC and the more advanced and size-separation instruments like SMPS, FMPS, and Nanoscan.

The results produced by use of the CPC shows the most variable results compared to the other instruments in this study. As shown in Table 3, the concentration measured by the CPC agree fairly well with the UFP concentration measured by SMPS in the PVC welding scenario (ratio 1.04), however, for the MIC welding scenario, the ratio CPC to SMPS is 6.05, and the CPC results exceed the remaining concentrations of UFP particles from all other instruments by more than a factor of two. Only for the MIC welding scenario, the ratio between the results are improved by evaluating the total particle size of the instrument, which is primarily caused by the particle size distribution of the particles measured by the SMPS show mainly particles above 200 nm. It seems as if the type of particles is important to understand the degree of agreement between CPC results and other results. The candle-burning scenario holds 99% ultrafine particles, as seen in Table 6. The lower range of the UFP interval for both Nanoscan and SMPS agree fairly well with the lower detection limit for the CPC, yet, the ratios are still 2.96 between CPC and SMPS and 1.91 between CPC and Nanoscan.

**Table 6:** Percentage UFP of the total particle measured by each instrument in each scenario

	PVC welding	Candle-burning	MIC welding	Cooking
SMPS	50	99	24	58
FMPS	71	99	23	76
Nanoscan	62	99	21	75

The results in this study is a laboratory investigation. The layout of the experimental setup included four tubing’s, one for each instrument. All tubes were assembled with a band ensuring that the aerosol collection took place at the same place. Particle level, however, can fluctuate within short distances, which could influence the results. The results is reported as 1 min averages in order to minimize the influence of fluctuations between the inlets of the four tubing’s.

### Concentration versus time profiles

The particle number concentration of UFP versus time profiles of the scenarios is illustrated in Fig. 2a, d. The correlation between results measured by the instruments is shown in Table 4. Evaluating the UFP results, the highest correlation was found between the Nanoscan and SMPS, with Pearsson correlation coefficients above 0.9 for all scenarios. The correlation between FMPS and SMPS is very good for both PVC welding and cooking with Pearson correlation coefficients above 0.96. For the candle-burning scenario, the correlation seems to be slightly lower, even that the very short period of lighting the candles with two matches and putting them out at the end of the experiment, not is included. For the MIC welding scenario, the decay of the concentration after each welding period seems to be slower for the FMPS instrument and, especially for the CPC, where the build-up of concentration also seems to be slower. No explanation was found for this.

The FMPS results did not decrease to zero between the MIC welding periods either, as illustrated in Fig. 2c. The FMPS is well known to be influenced by fouling of the instrument during periods with high concentrations, but this is probably not the reason; primarily because of the use of a dilutor before the instrument, and also, this would affect all measurements, not just the decay period. The particles that are measured in between the welding periods are mainly particles with diameters at midpoint of 9.8 and 10.8 nm. This could be particles originating from the background air in the lab, however, both Price, et al. [35] and Jeong, et al. [25] discuss whether this could be an erroneous FMPS peak.

For the CPC, the concentration versus time profiles show that the measurement differs in many ways. For the PVC welding scenario (Fig. 2a), the measurement shows a deviating time profile, not showing the peaks nor the decay during the experiment, only the decay after the end of experiment, and also with a slower decay than the other instruments. No explanation is found for this difference, which is also reflected in low Pearsson correlation coefficients between CPC and SMPS results.

### Particle size distribution

The lowest particle size measured varied by the instruments, as shown in Table 2. This influenced the results for the scenarios where a high number of very small particles was produced. The ratios between the FMPS and SMPS results varied between being over and under 1. For MIC welding and candle-burning, the UFP ratio was 2.65 and 3.31 for the UFP fraction, while the UFP ration for PVC welding and cooking was 0.75 and 0.83. The FMPS measures a small size range below the SMPS and the Nanoscan (5.6 nm-10/13.3 nm); if nanoparticles are produced in this small interval, the FMPS should measure a higher concentration, which is the explanation for the higher concentrations for the candle-burning scenario, as indicated by Fig. 2d.

The particle size distribution in the MIC scenario found by Nanoscan and the FMPS differ from the SMPS results (Fig. 1c). The small peak shown by the FMPS at 9.8 nm is discussed above. FMPS showed a maximum of particles at 165 nm, the Nanoscan at 205 nm, while SMPS showed a maximum of 310 nm particles. Lehnert, et al. [36] have shown particle distributions found by different types of welding, unfortunately, not MIC, but tungsten inert welding (TIG), flux-cored arc welding, (FCAW), and gas metal arc welding (GMAW) and shielded metal arc welding (SMAW). The results by Lehnert showed the same type of particle size distribution as Nanoscan and FMPS in this study, with mainly unimodal particle size distributions [36]. The particle size distribution found by the SMPS was analysed for all single measurements and the particle size distribution was unchanged within the entire experiment. No explanation was found for the dip in concentration for 191 m, the particle with a subsequent maximum of 310 nm.

It is well known that the agglomeration nature of particles measured could influence the results. Zimmermann et al. have shown that FMPS substantially overestimate particle number concentration in the 20–120 nm range when measuring diesel exhaust particles due to this phenomenon [37]. No corrections due to agglomeration is performed in this study of any of the equipments used. Use of agglomeration correction of the FMPS results could probably have improved the correlation to the SMPS results.

### Instrument comparison

One of the main differences between the instruments is the particle size range. A comparison can be made with small size ranges that fits optimally between the instruments, and this would have been done if this study was a general comparison of the instruments. For the purposes of discussion, a more practical approach, to compare the ability of four instruments to quantify the number concentration of particles below 100 nm, the particle size range was compared, as all particles quantified in the size range below 100 nm, without consideration to the differences in the lower particle range.

SMPS is considered the best technology commercially available to measure particle size distributions [30], and is often mentioned as a reference instrument for measurements within the nano-range. However, it is also an advanced instrument, not ideally suited for monitoring the workplace [23]. The SMPS has the highest resolutions in channels among the instruments tested (64 channels), and is suitable for a large range of applications. The instrument is, however, the most complicated to operate, with a wide range of various adjustments due to scan time, sheath air flow, impactor used, type of Differential Mobility Analyzer (DMA), etc. Yet, the most important challenge is that it is large and power demanding, and thus, difficult to use in field studies within occupational hygiene. Situations with mobile workplaces and irregular exposure or occasional exposure might be difficult to evaluate with the use of stationery equipment. The SMPS instruments contain a radioactive source, either ^85^Kr or an X-ray, which require permission from radiation protection authorities.

The FMPS instrument is equipment that is simpler to operate than the SMPS. The instrument has a single setup, which makes it easier in practical use. The main advantage is the high time resolution. The FMPS measures using a separate measurement each second, and thus, is the most suitable for catching short-time variations. However, the quantification of concentrations may, in some cases, differ from the SMPS results, at least when including a considerable number of particles detected by the FMPS at 10 nm, which is below the detection limit of the SMPS. In this study, the 10 nm particles measured for MIC welding could be an erroneous peak, while the 10 nm particles measured from candle-burning is likely correct, as it was also confirmed by Manoukian, et al. [2013], who found that, during burning, only one mode was observed below 11 nm [38].

A comparison of FMPS with a SMPS installed with a nano-DMA is recommended to compare the lower end of the particle size range in detail. No setup of the SMPS allows for direct comparison with the entire measurement range of the FMPS. A nano-DMA allows for measurements in the 2–150 nm range, while the best fit between the optimal fit for the long DMA was tested in this study (13.3–487 nm). Jeong and Evans [2009] have proposed an empirical correction procedure to consider the discrepancy between FMPS and SMPS for ambient particle studies [25], subsequently confirmed by Zimmerman, et al. [37] The correction, however, is not useful for all datasets, as mentioned by Ashbach, et al. [24], as Jeong, et al. observed an underestimation of the number concentration by the FMPS compared to the SMPS.

The CPC instrument is small and portable, probably the most widely used instrument, and is easily used for occupational hygiene applications. However, the results showed that the CPC 3007 did not show comparable results with the remaining instrument and quantification of exposure in a workplace with a subsequent comparison with nano reference values is not recommended;. For more practical purposes, the instrument could be used for indicating the existence of submicrometre particles, but not for a more detailed study, which was also discussed by Leskinen [23]. An important aspect of the CPC is that the maximum range of concentrations measured by the instrument is 1 × 10^5^ particles/cm^3^. Anything above this concentration may be coincidental [2]. Within occupational hygiene studies, concentrations above 10^5^particles/cm^3^ are often found [5], which emphasises that the CPC instrument has limited utility for this type of study.

The results by the Nanoscan SMPS is found to have the highest correlation with the SMPS among the instruments tested, regardless if the UFP fraction or the total particle size range is compared. The results tend, however, to overestimate the concentrations compared to the SMPS results for all tested scenarios within the same range, as shown by Stabile [30] and Foncesa [33]. The instrument is easy to use, lower in weight, and not power demanding, which are required for occupational hygiene measurements [2, 5, 23]. There is a need for workplace comparisons between Nanoscan measurements and SMPS measurements to determine if the Nanoscan overestimates the results compared to the SMPS, in general, or if this depends on type of particles investigated.

Van Broekhuize and Dorbech-Jung [2013] conducted a small pilot study with nanomaterial using industries in the Netherlands and found that most companies working with nanomaterials accept NRVs as tools to minimise possible adverse health effects among employees [39]. The scenarios tested in this study are not relevant for comparison with future reference values, however, based on the results of this study, the choice of measurement equipment could be crucial when evaluating measurement results against a reference value of 40,000 particles/cm^3^. The National Institute for Occupational Safety and Health (NIOSH) has recommended a time-weighted average exposure limit for ultrafine titanium dioxide (TiO2).

In a test chamber study, West, et al. [40] demonstrated a potential for overexposure to nano-TiO2 during a common construction task. This stressed the need for method development, standardisation, and harmonisation of particle sampling protocols, further recommended by Price, et al. [35]

## Conclusion

The results of this study show that large variations between nanoparticle measurements and results exist for the four scenarios tested. The SMPS results are used as reference for quantification of the concentrations measured.

It is important that results are reported accurately. Results expressed as the concentration of ultrafine particles must be calculated from a size range containing only ultrafine particles and not a bulk measurement of all particles in a larger range. The only exception would be if the size distribution is known in advance and it is well established that the aerosol contains only particles in the ultrafine range. Instead, concentrations measured by bulk-measuring instruments should be reported as concentrations of submicrometre particles.

CPC 3007 did not show comparable results with the remaining instruments. The results by the Nanoscan SMPS were found to have the highest correlation with the SMPS among the instruments tested, regardless if the UFP fraction or the total particle size range is compared. The Nanoscan results tends to overestimate the concentrations measured by the SMPS, regardless if UFP concentration or total concentration is compared. The ratio between the UFP results vary between 1.44 and 2.01 and the total concentration between 1.18 and 2.33 is within the same range as shown by Stabile [30] and Foncesa [33]. A more detailed study according to occupational hygiene applications is required for the FMPS instrument. Differences in particle size distribution, concentration quantification, and the need for a correction procedure for FMPS results over SMPS results should be investigated further.

Based on the results of this study, the choice of measurement equipment could be crucial when evaluating measurement results against a reference value or a limit value for nanoparticle exposure. This stresses the need for method development, standardisation, and harmonisation of particle sampling protocols. Until this is established, the SMPS instrument is the most reliable for quantification of the concentrations of UFP. However, in a more practical occupational hygiene context, the Nanoscan SMPS should be further tested.

## Data Availability

The datasets used and analysed during the current study are available from the corresponding author on reasonable request.

## Abbreviations

AM: Arithmetic mean
CPC: Condensation particle counter
DEHS: Di-ethyl hexyl sebacate
DMA: Deifferential mobility analyser
dNdlogDp: Normalised concentration
EC: Electrostatic classifier
FCAW: Fluc cored arc welding
FMPS: Fast Mobility Particle Sizer
GDC: Geometric standard deviation
GM: Geometric mean
GMAW: Gas metal arc welding
IQR: Interquartile range
MIG: Metal inert gas
NIOSH: Institute for Occupational Safety and Health
NRV: Nano reference values
PVC: Polyvinyl chloride
SD: Standard deviation
SMAW: Shielded metal arc welding
SMPS: Scanning Mobility Particle Sampler
TIG: Tungsten inert welding
UFP: Ultrafine particles
